# Current Therapeutic Strategies for FLT3-Mutated Acute Myeloid Leukemia: A Narrative Review

**DOI:** 10.7759/cureus.95338

**Published:** 2025-10-24

**Authors:** Lainee Swanson, Jonathan Sharp, Aliena Liaw, Delbert Abi Abdallah, David M Duriancik

**Affiliations:** 1 Medicine, Lake Erie College of Osteopathic Medicine (LECOM), Erie, USA; 2 Biology, Allegheny College, Meadville, USA; 3 Medicine, St. John's Riverside Hospital, Yonkers, USA; 4 Microbiology, Duquesne University College of Osteopathic Medicine, Pittsburgh, USA; 5 Biochemistry, Lake Erie College of Osteopathic Medicine (LECOM), Erie, USA

**Keywords:** acute myeloid leukemia, cell lines, fms-like tyrosine kinase 3, midostaurin, quizartinib

## Abstract

Patients with acute myeloid leukemia (AML) typically present with unexplained weight loss, fatigue, and other nonspecific and varied symptoms, and are diagnosed by the aggressive onset of symptoms and a peripheral blood smear with a high proportion of myeloblasts. Patients with AML may have a variety of mutations, but mutations in FMS-like tyrosine kinase 3 (FLT3) are prognostic of outcomes. Notably, only four human AML cell lines express an internal duplication in FLT3, while two human AML cell lines contain an activating mutation in the juxtamembrane domain. A variety of other cell lines express mutated FLT3, including lymphoid leukemia cell lines. Several drugs have been developed to target mutated FLT3, but only three have been FDA-approved. In this review, we summarize the human myeloid leukemia cell lines that express mutated FLT3 and the effect of several drugs on these cell lines. Our aim in this review is to provide clinicians with a basic science understanding of human myeloid leukemia cell lines and to provide scientific researchers with the clinical implications of FLT3 signaling inhibition.

## Introduction and background

Myeloid leukemia is a rare cancer accounting for approximately 1% of all cancers and 11,500 deaths per year in the US [1]. In 2020, it was estimated that 20,000 new cases of myeloid leukemia were diagnosed, with a peak incidence at 65 years of age [2]. Myeloid leukemia consists of a heterogeneous group of malignancies presenting as an acute or chronic disease with expansion of distinct hematopoietic progenitor cell populations. Patients with chronic myeloid leukemia (CML) present with less than 20% myeloblasts in a peripheral blood smear and a slower onset of symptoms [3]. The most common genetic cause of CML is the reciprocal translocation and fusion of the breakpoint cluster region on chromosome 22 with the Abelson gene on chromosome 9, resulting in the Philadelphia chromosome [4]. Patients with acute myeloid leukemia (AML) present with greater than 20% myeloblasts in a peripheral blood smear and a more aggressive onset of symptoms [5]. Patients with AML typically present with weight loss, loss of appetite, fatigue, fever, night sweats, and other nonspecific symptoms. A complete blood count, peripheral blood smear, and bone marrow aspiration and biopsy simultaneously confirm a diagnosis and classify the AML subtype [6]. Recently, mutations in FMS-like tyrosine kinase 3 (FLT3) have been shown to be prognostic of AML outcomes. Approximately 30% of AML patients have a mutation in FLT3 resulting in a constitutively active receptor. The FLT3 mutation, resulting in AML, may be due to an internal tandem duplication (FLT3-ITD) of varying length and location or a point mutation in the tyrosine kinase domain (FLT3-TKD). Several research groups estimated that approximately 20% of AML patients have an FLT3-ITD mutation [7-10], while Fernandez et al. estimated approximately 25% have the FLT3-ITD mutation [11]. The FLT3 mutation may also be due to a point mutation in the FLT3-TKD, which was estimated to be present in 7%-10% of patients with AML [11-13].

In this review, we summarize human myeloid leukemia cell lines expressing FLT3 mutations and current therapies for FLT3-mutant AML. Several other reviews have been published on FLT3 signaling in normal and malignant conditions [14,15], but this narrative review focuses on the available FLT3-mutated AML cell lines. In this review, we aim to provide clinicians and scientific researchers with a basic science understanding of human myeloid leukemia cell lines and an understanding of the implications of FLT3 signaling inhibition.

## Review

Human cell lines expressing mutant FLT3

There are several human myeloid leukemia cell lines available, but only a few contain activating mutations in FLT3. Quentmeier et al. analyzed 69 different human myeloid leukemia cell lines for the expression of mutated FLT3 [16]. Of the cell lines analyzed, only two were homozygous (MUTZ-11 and MV-4-11) and three were heterozygous (MOLM-13, MOLM-14, and PL-21) for the FLT3-ITD mutation. FLT3 signaling in the MUTZ-11, MOLM-13, and PL-21 cell lines was not constitutively active, while signaling in the MV-4-11 cell line was constitutive and independent of the presence of FLT3L [16]. Previous work done by Yokota et al. used different polymerase chain reaction (PCR) primers spanning the transmembrane domain and part of the juxtamembrane domain (exon 10) and a second set of primers specific for exon 11 that codes for the juxtamembrane domain and part of the tyrosine kinase domain [10]. Using these primers, MOLM-13 cells were found to have a mutation in the juxtamembrane domain of FLT3, while PL-21 cells did not have an FLT3 mutation. The juxtamembrane domain FLT3-ITD mutations were later mapped to exons 14 and 15 [12]. Quentmeier et al. used different PCR primers specific for FLT3-ITD in exons 14 and 15, as well as primers specific for FLT3-TKD in exon 20 [16]. Sequencing of the PCR product of the FLT3 mutated allele from PL-21 cells revealed that the ITD begins in intron 14 and spans the exon-intron junction [16], which likely explains the discrepancy between these publications and publicly accessible RNA sequencing data. We investigated publicly accessible RNA sequencing data [17,18] available from 70 cell lines with FLT3 mutations (Table 1).

**Table 1:** Leukemia cell lines expressing FLT3 mutations ^1^French-American-British classification of AML subtype ^2^Data obtained from DepMap Portal Dataset DepMap Public 25Q2. The database was searched for FLT3 mutations. Subsequently, the DepMap Public 25Q2 Dataset was analyzed for expression and copy number of myeloid and lymphoid cell lines with mutated FLT3 ^3^MONO-MAC-1 and MONO-MAC-6 Flt3 mutations were characterized as likely pathogenic FLT3-ITD: MS-like tyrosine kinase 3 internal tandem duplication; AML: acute myeloid leukemia; N/A: not available

Cell line	AML subtype of cell line^1^	Protein change^2^	Expression, Log_2_ (TPM + 1)^2^	Copy number^2^
FLT3-ITD in-frame insertions				
MOLM-13	M5: acute myeloblastic	E598_Y599insFDFREYE	7.00	1.29
MOLM-14	M5: acute myeloblastic	E598_Y599insFDFREYE	5.37	1.27
MV-4-11	M5: acute differentiated monocytic	D600_L601insHVDFREYEYD	6.51	1.00
KASUMI-6	M2: acute myeloblastic leukemia	G583_Y599dup	6.93	1.29
FLT3 point mutations				
HB1119	B-lymphoblastic leukemia	D835H	7.75	1.02
CTV1	Adult T-cell leukemia	G831E	N/A	N/A
CTV1DM	Adult T-cell leukemia	G831E	0.35	1.16
DAUDI	Burkitt lymphoma	G757E	1.45	1.01
CCRFCEM	T-lymphoblastic leukemia	A627T	N/A	1.00
MONO-MAC-1^3^	M5: acute differentiated monocytic	V592A	8.10	1.35
MONO-MAC-6^3^	M5: eosinophilic/monocytic	V592A	9.01	3.63
Nonactivating FLT3 point mutations				
TF-1	M6: erythroleukemia	E964G	0.00	0.91
FARAGE	Germinal center B-cell type	D62A	1.94	1.03
ALLPO	B-lymphoblastic leukemia	V491L	N/A	1.20
WSUDLCL2	Diffuse large B-cell lymphoma	L168F	N/A	1.27

Seven acute myeloid cell lines were positive for FLT3 mutations in the DepMap portal database. A total of eight lymphoid leukemia cell lines were positive for a mutation in FLT3, including one T lymphoblastic leukemia, two B lymphoblastic leukemia, two mature T and NK neoplasms, and three mature B cell neoplasms. The remaining 55 cell lines containing FLT3 mutations were a collection of epithelial cancer cell lines from a variety of tissues. This analysis provides evidence that FLT3 mutations may not only be relevant in hematopoietic malignancies but also in various epithelial malignancies from a variety of different tissues, which were beyond the scope of this review.

The MONO-MAC-1, MONO-MAC-6, and TF-1 cell lines were found to contain FLT3 mutations in the DepMap portal; however, when analyzed by Quentmeier et al. [16], they were not described as having a mutation in FLT3. The TF-1 cell line mutation in FLT3 was described as a missense mutation resulting in a glutamic acid to glycine change at amino acid 964 in the cytoplasmic domain, which may explain the discrepancy between Quentmeier et al. and data from the DepMap portal. Interestingly, the MONO-MAC-1 and MONO-MAC-6 cell lines were characterized as having a “likely pathogenic” FLT3 point mutation in the juxtamembrane domain, and the FLT3 copy number of MONO-MAC-6 cells was increased compared to other cell lines [19]. The KASUMI-6 cell line contains a duplication in the juxtamembrane domain, but was not analyzed by Quentmeier et al., though the authors did analyze KASUMI-1 and KASUMI-3 cell lines and did not find an FLT3-ITD mutation. The RNA sequencing data from the MUTZ-11 cell line were not currently available on the DepMap portal. Therefore, analysis of the data from the DepMap portal was generally consistent with data from Quentmeier et al., but heterozygosity and dependence on FLT3L could not be determined from RNA sequencing analysis available from the DepMap portal.

The human AML cell lines expressing mutant FLT3 were derived from diverse patients with varying cytogenetics. The MONO-MAC-1 and MONO-MAC-6 cell lines were derived from the same 64-year-old male patient diagnosed with AML M5 at relapse [20]. The karyotype of MONO-MAC-1 and MONO-MAC-6 cells were shown to be similar as 48, XY, +8, +13, +18, +21, -17, -20 add(3)(q27), t(9;11)(p13;q23), t(10;13)(q23.3;q13), t(12;17)(q21;q11), t(21;21)(q22.1;q11/22) with MONO-MAC-6 having double chromosome number [21] and cytogenetics were positive for KMT2A-MLLT3 fusion gene and a mutation in TP53 DNA binding domain [22,23]. The MOLM-13 and MOLM-14 cell lines were derived from the same 20-year-old male patient diagnosed with AML M5a at relapse [24]. The karyotype was described as 51, XY, +8, +8, +8, +13, ins(11;9)(q23;p22;q23) for MOLM-13 cells and 49, XY, +6, +8, +13, ins(11;9)(q23;p22;q23) for MOLM-14 cells and cytogenetic analyses were positive for KMT2A-MLLT3 fusion gene in both cell lines [24]. Interestingly, the MOLM-13 and MOLM-14 cell lines contain an extra copy of chromosome 13, and the location of the human FLT3 gene has been mapped to chromosome 13q12.2. It is unclear if the additional chromosome 13 in these cells is positive for wild-type FLT3 or the FLT3-ITD. Unlike the sister cell line pairs of MOLM-13 and MOLM-14, as well as MONO-MAC-1 and MONO-MAC-6, the KASUMI-6 cell line was not derived from the same patient as the KASUMI-1 or KASUMI-3 cell lines. The KASUMI-6 cell line was established from a 64-year-old man diagnosed with AML M2 at relapse [25]. The karyotype of KASUMI-6 cells was described as 45, XY, -9, add(12)(p11), add(13)(p11) with cytogenetics revealing mutations in CEBPA and TP53 [25,26]. The MV-4-11 cell line was derived from a 10-year-old man diagnosed with biphenotypic B-myelomonocytic leukemia [27]. The MV-4-11 cell line is, to our knowledge, the only human AML cell line that is homozygous for the FLT3-ITD mutation. The signaling of FLT3 independent of FLT3L remains controversial [16,19]. The karyotype of MV-4-11 cells was described as 48, XY, t(4;11)(q21;q23), +8, +19, and cytogenetics were positive for a KMT2A-AFF1 fusion gene [27]. Interestingly, the JIH5 cell line was reported in DepMap as having an FLT3-FLT3 fusion protein highly expressed at 10.99 fusion fragments per million [17,18]. However, this is likely an artifact. The JIH5 cell line was recently established from a 21-year-old woman at second relapse with acute leukemia of ambiguous lineage [28]. The karyotype of JIH5 cells was described as 46, XX, t(4;5)(q35;q35), t(5;8)(q32;q22), t(7;21)(p15;q21, t(12;22)(p13;q13), del(2)(q33)t(2;2), der(6)del(6)(p21;p22)t(6;10)(p23;q23), der(9)del(9)(p21)del(9)(q34.2), der(10),t(6;10), der(17)t(17;17)(p13;q22), del(19)(q13) with 10 copy number alterations and nine somatic mutations [28]. However, in direct relation to FLT3 expression, Zhao et al. [29] reported that the ZNF384 fusion (most commonly with EP300) dramatically increases expression of wild-type FLT3 and was sensitive to FLT3 inhibitors (gilteritinib and lestaurtinib). Fusion proteins of ZNF384 appeared to bind to the enhancer region of FLT3, causing overexpression through chromatin remodeling. The cell lines with mutated FLT3 were derived from patients with various AML subtype diagnoses and have additional genetic abnormalities that also contribute to their pathogenicity but do not appear to alter their sensitivity to inhibitors of FLT3.

Current therapies for AML with mutant FLT3

Treatment of AML is dependent on whether the patient is newly diagnosed or previously untreated, in remission, or in relapse. Newly diagnosed or previously untreated AML patients, regardless of AML genotype or phenotype, are started on an induction therapy with the goal of complete remission (CR). The current standard induction therapy includes cytarabine, a nucleoside analog and antimetabolite, administered at 200 mg/m^2^ on days one through seven, plus the type II topoisomerase inhibitor daunorubicin, administered at 60 mg/m^2^ on days 1-3 (7 + 3 cytarabine and daunorubicin). The treatment may last for 28 days, during which two treatment cycles may be administered. Remission is defined as an absolute neutrophil count greater than 1,000/mm^3^, a platelet count greater than 100,000/mm^3^, a normocellular marrow with less than 5% blasts, and absence of symptoms [5]. If induction therapy is successful, patients become candidates for allogeneic hematopoietic stem cell transplantation. In patients diagnosed with FLT3-mutated AML, there is evidence that FLT3 inhibitors are an effective adjunctive therapy intended to induce CR, progression to allogenic hematopoietic stem cell transplantation, and remission maintenance after transplantation. Kennedy and Smith provide an extensive review of current therapies for mutated FLT3 as well as future directions, which were beyond the scope of this review [30]. Here, we provide a brief overview of therapies for mutated FLT3, focusing on the effects of these inhibitors on mutated FLT3 AML cell lines.

Currently, many types of tyrosine kinase inhibitors are used in the treatment of FLT3-mutated AML. These FLT3 inhibitors include midostaurin, sorafenib, gilteritinib, crenolanib, quizartinib, FF-10101, SEL24-B489, TAK-659, and others [11]. Three FLT3 inhibitors are currently FDA-approved for patients with AML: midostaurin for use in newly diagnosed FLT3-mutated AML, gilteritinib for use in relapsed or refractory FLT3-mutated AML, and quizartinib for induction, consolidation, and maintenance monotherapy. Midostaurin and sorafenib are first-generation, broad-spectrum multikinase inhibitors that are nonspecific for mutated FLT3. Second-generation inhibitors, such as gilteritinib, crenolanib, and quizartinib, are more specific and potent inhibitors of FLT3 [31], resulting in less toxicity and fewer harmful side effects [32]. Inhibitors that target the active conformation of FLT3 are type I inhibitors and are effective for AML patients with either FLT3-ITD or FLT3-TKD, while type II inhibitors target the inactive conformation of FLT3 and are effective only for AML patients with FLT3-ITD mutations. Both type I and type II inhibitors function near the ATP-binding domain, but type I inhibitors may also function in the activation loop [33]. Midostaurin, a first-generation type I tyrosine kinase inhibitor, induced apoptosis (Table 2) of MV-4-11, MOLM-13, and MONO-MAC-6 cells [34,35]. Midostaurin was FDA-approved in 2017 for newly diagnosed FLT3-positive AML and has been incorporated into the recommended treatment regimen. Midostaurin therapy, in combination with 7 + 3 cytarabine and daunorubicin induction and consolidation therapy, improved median survival from 26 to 72 months. However, CR rates and disease-free survival rates were similar between therapies with or without midostaurin in the Randomized Adjuvant Treatment for Initial FLT3-mutated Young adults with AML (RATIFY) trial (NCT00651261). Long-term maintenance doses of chemotherapy did not benefit AML patients in remission. However, the addition of midostaurin or oral azacitidine to maintenance chemotherapy did have a benefit [36]. The RATIFY trial was not designed to assess maintenance therapy, as it was confined to the subgroup of patients who had achieved CR post-induction and consolidation. Consequently, while midostaurin is recommended for AML maintenance therapy in the European Union, it is not approved for such use in the United States [37].

**Table 2:** Cell line sensitivities to FLT3 inhibitors ^1^Only the MV-4-11, MOLM-13, and MONO-MAC-6 cell lines were available in these datasets. MONO-MAC-1, MOLM-14, and KASUMI-6 cell lines were not available ^2^Data acquired from GDSC1 performed by Wellcome Sanger Institute and Massachusetts General Hospital between 2009 and 2015 using Syto60 or Resazurin with media alone as the control ^3^Gilteritinib and crenolanib data were not available ^4^Data acquired from GDSC2 performed by Wellcome Sanger Institute and Massachusetts General Hospital post-2015 using CellTiter-Glo with media containing DMSO as the control. Only sorafenib and venetoclax had data available from GDSC2 (MV-4-11, MOLM-13, and MONO-MAC-6), all other data were from only GDSC1 GDSC1: Genomics of Drug Sensitivity in Cancer dataset 1; N/A: Not available; GDSC2: Genomics of Drug Sensitivity in Cancer dataset 2

Drug	Cell line^1^		
	MV-4-11	MOLM-13	MONO-MAC-6
Midostaurin^2^	21.1 nM	18.8 nM	98.5 nM
Gilteritinib^3^	N/A	N/A	N/A
Quizartinib^2^	1.1 nM	0.9 nM	4.0 nM
Sorafenib^2,4^	4.0-4.3 nM	4.2 nM	10.4-31.4 nM
Crenolanib^3^	N/A	N/A	N/A
Venetoclax^4^	1.8 nM	3.1 nM	1.6 uM

Gilteritinib, a second-generation type I inhibitor, was not available in the Genomics of Drug Sensitivity in Cancer database, but had a reported IC50 value of approximately 1.5-12 nM for cells with various FLT3 mutations and wild-type FLT3 [38]. Gilteritinib inhibited the proliferation of MV-4-11 and MOLM-13 cells with IC50 values of 0.92-3.3 and 2.9-19.0 nM, respectively [32,39]. Gilteritinib was tested on MV4-11 and MOLM-13 cell lines; the inhibitor was effective against ITD and D835 point mutations by decreasing FLT3 phosphorylation levels [18,19]. Off-target inhibition of Axl and c-KIT occurs at much larger IC50 values of approximately 40-100 nM, respectively [38]. On November 28, 2018, the FDA approved gilteritinib for relapsed or refractory FLT3-positive AML. Before starting therapy, it is recommended to regularly monitor blood counts, serum chemistries, and creatine phosphokinase levels. Due to the potential for a prolonged QT, an echocardiogram should also be performed prior to treatment, on days 8 and 15 of the first chemotherapy cycle, and prior to the start of the subsequent two cycles.

Quizartinib is a second-generation type I inhibitor that has a reported IC50 value of 0.56 nM and exhibits high selectivity at low concentrations for FLT3, KIT, colony-stimulating factor-1 receptor, platelet-derived growth factor (PDGF) receptor, and RET kinase. Quizartinib induced apoptosis of MV-4-11, MOLM-13, and MONO-MAC-6 cells (Table 2) at IC50 values of 1, 1, and 4 nM, respectively [34,35]. Quizartinib was examined as an FLT3 inhibitor in various experimental modalities, including cell lines, primary blast samples, and murine xenograft models. In tests involving MV4-11 or MOLM-14 cells and blasts obtained from individuals with FLT3-ITD AML, quizartinib induced apoptosis at IC50 values ranging from 1 to 2 nM [40]. On July 20, 2023, the FDA approved the use of quizartinib with the standard 7 + 3 cytarabine and anthracycline induction, consolidation, and maintenance monotherapy following consolidation chemotherapy for FLT3-ITD-positive AML patients. The decision to either maintain or raise the dosage is contingent upon correcting the absolute QT interval for heart rate using Fridericia's formula.

Sorafenib is a first-generation type II multikinase inhibitor with preclinical efficacy against RAS/RAF, c-KIT, vascular endothelial growth factor receptor, PDGF receptor kinases, and FLT3. Sorafenib induced apoptosis (Table 2) of MV-4-11 with an IC50 of 5nM, MOLM-13 with an IC50 of 5nM, and MONO-MAC-6 with an IC50 of 10 - 30nM [34,35]. It is currently approved by the FDA for hepatocellular carcinoma, renal cell carcinoma, and differentiated thyroid carcinoma, but not AML with FLT3 mutations. The randomized placebo-controlled SORAML trial showed that for patients with newly diagnosed AML, sorafenib led to significant improvement in event-free survival (EFS) and relapse-free survival (RFS) rates. The five-year EFS rate was 41% vs. 27% in the placebo group, and the five-year RFS rate was 53% vs. 36% in the placebo group. However, there was no statistical significance in the improvement of overall survival (OS) rate in patients treated with sorafenib, demonstrated by the five-year OS of 61% vs. 53% in the placebo group [41]. These results do not support the standard use of sorafenib in intensive first-line treatment for patients with AML. SORMAIN, a randomized, placebo-controlled, double-blind phase II trial, studied the effect of sorafenib in patients with complete hematologic remission after hematopoietic cell transplant (HCT). The findings indicated that the use of sorafenib as a maintenance treatment decreases the likelihood of relapse and mortality following HCT for FLT3 ITD-positive AML, with a 24-month RFS rate of 85%, compared to 53.3% in the placebo group [42]. These findings indicate that while sorafenib may not be efficacious as an intensive initial treatment, its utilization as maintenance therapy following allogeneic stem cell transplantation or in conjunction with azacitidine or cytarabine in cases of relapse could be justified [41]. The Acute Leukemia Working Party of the European Society for Blood and Marrow Transplantation suggests posttransplant maintenance therapy with sorafenib. This maintenance therapy should begin at the onset of hematologic reconstitution and persist for a minimum of two years, contingent on individual tolerance [43].

Crenolanib is a second-generation type I inhibitor with activity against PDGFR-beta, FLT3-ITD, and FLT3-TKD mutations. Crenolanib data were not available in the Genomics of Drug Sensitivity in Cancer database, but induced apoptosis of MV-4-11 and MOLM-13 cells with an IC50 of approximately 1 and 5 nM, respectively [44]. Crenolanib demonstrated promising results as a single agent in R/R FLT3-positive AML and in combination with chemotherapy [45]. Crenolanib is not FDA-approved for the treatment of AML, but there are two phase III trials that are actively recruiting at the time of writing this article. One is studying the effect of crenolanib vs. midostaurin following induction chemotherapy and consolidation therapy in newly diagnosed FLT3-mutated AML (NCT03258931). The goal of this study is to investigate the primary outcome measure of EFS rates in five years and secondary outcomes measuring seven-year OS and five-year RFS rates, composite CR rate, and duration of response. The other clinical trial (NCT03250338) is investigating the efficacy of crenolanib with chemotherapy vs. chemotherapy alone in R/R FLT3-mutated AML. The goal of this study is to investigate the primary outcome measures of EFS rates in three years and secondary outcomes measuring OS, RFS, CR, and measurable residual disease-negative CR rates in three years.

Drugs in development to overcome resistance to FLT3 inhibition

Despite clinical efficacy upon initial exposure to FLT3 inhibitors, resistance to inhibition can develop. Resistance mechanisms and implications have been thoroughly reviewed elsewhere [30,46]. Resistance mechanisms include secondary mutations in FLT3, including the F691L mutation [47,48], mutations in cell signaling proteins such as genes involved in the RAS pathway, TP53 and IDH1/2 [49,50], or extrinsic mechanisms such as changes in bone marrow stromal cells that increase the metabolism of FLT3 inhibitors [51] or other signaling proteins [52,53]. Interestingly, FLT3L was also increased in the presence of FLT3 inhibitors, leading to activation of wild-type FLT3 and resulting in the development of resistance to FLT3 inhibitors [52,54]. In mice and humans, B lymphocytes and plasmacytoid dendritic cells can be expanded with exogenous FLT3L [55,56]. Rickmann et al. reported an increased and highly variable frequency of both myeloid and plasmacytoid dendritic cells in AML patients with FLT3-ITD compared to AML patients without the FLT3-ITD mutation. The function of these myeloid and plasmacytoid dendritic cells from patients with the FLT3-ITD mutated AML was also highly variable and abnormal compared to healthy donors [57]. At this time, the changes in dendritic cell frequency and function are interesting observations, but the clinical implications of these changes require further investigation. To overcome some resistance mechanisms, drugs that target both FLT3 signaling and parallel signaling cascades are in development and include SEL24-B489 (dual inhibition of FLT3-ITD and PIM kinases), TAK-659 (dual inhibition of FLT3 and spleen tyrosine kinases, SYK), and FF-10101 (irreversible, covalent FLT3 inhibitor). Using AML cell lines would help to further elucidate mechanisms of secondary mutations in FLT3 and signaling cascades, but the utility of cell lines in studying the effects of the bone marrow stroma would be limited. Due to possible resistance mechanisms as well as changes in immune cell phenotype and function, patients treated with FLT3 inhibitors during maintenance chemotherapy should be monitored for viral infections and other complications.

FF-10101 is an irreversible, type I covalent inhibitor of FLT3 that has exhibited activity against R/R AML, including those with activating FLT3-ITD mutations resistant to gilteritinib, quizartinib, and other FLT3 kinase inhibitors. The resistance is secondary to FLT3 mutations such as F691L, Y842C/H, and D835Y mutations [58]. The function of FF-10101 is dependent on a single covalent bond located at C695. Its favorable resistance profile may contribute to improved single-agent efficacy for patients with FLT3-positive AML. FF-10101 induced a higher rate of apoptosis in MOLM-14 cells in HS5 conditioned media compared to gilteritinib at comparable doses ranging from 50-100 nM for each drug. Additionally, FF-10101 demonstrated strong inhibitory effects on the growth of human AML cell lines containing FLT3-ITD, such as MOLM-14 and MV4-11. It also exhibited effectiveness against various mutant FLT3-expressing 32D cells, including quizartinib-resistant mutations located at D835, Y842, and F691 residues in the FLT3 kinase domain. FF-10101 inhibited the growth of primary AML cells containing either FLT3-ITD or FLT3-D835 mutation, both in vivo and in vitro. These data suggest that FF-10101 could serve as an effective second-line TKI for FLT3 mutant AML patients who relapse with known resistance-conferring FLT3 TKD mutations. This is especially true in situations where FF-10101 exhibits greater potential to overcome resistance to TKI than gilteritinib, particularly at submaximal plasma concentrations. However, FF-10101 frequently demonstrates higher cytotoxic effects than gilteritinib at comparable dosage levels in AML cell lines, potentially due to the suppression of extracellular signal-regulated kinase reactivation after prolonged drug exposure. These findings suggest that FF-10101 holds promise as a therapeutic agent for individuals with FLT3 AML, including those with activation loop mutations recognized clinically as quizartinib-resistant mutations; however, further investigation is required [59].

Mivavotinib (TAK-659) is a reversible, investigational, type I tyrosine kinase inhibitor with dual activity against SYK and FLT3. Incubation of MOLM-14 cells with mivavotinib reduced FLT3 phosphorylation at an IC50 value of 80 nM [60]. A phase Ib study investigating the safety, tolerability, and efficacy of mivavotinib in patients with R/R AML was conducted, and findings show modest mivavotinib activity in R/R AML. The studied doses of mivavotinib were 140 mg daily or 60 mg twice daily. In the study, 70% of participants encountered bleeding incidents (grade 1 or 2), which were determined to be unrelated to the study treatment and resolved without requiring a dose adjustment. Approximately 26% of participants experienced more severe bleeding events of grade 3 or higher, with the highest frequency observed in those receiving the 80 mg dose twice daily. The study suggested that these bleeding events might have been influenced by various confounding factors, including the AML disease status of the patient, concurrent thrombocytopenia, and elevated doses of mivavotinib. Given the potential risk of bleeding, any subsequent clinical exploration of mivavotinib should be approached with caution. Currently, there are no active clinical trials for mivavotinib [60].

Signaling of FLT3 activates PIM, and SEL24-B489 is a type I, dual PIM and FLT3 inhibitor. SEL24-B489 binds to and inhibits PIM-1, PIM-2, and PIM-3 as well as mutant forms of FLT3, which may interrupt the G1/S phase of cell cycle and cell proliferation, inducing apoptosis in tumor cells. PIM kinases are considered key contributors to the resistance phenotype and blocking them in relapsed samples re-establishes cellular responsiveness to FLT3 inhibitors. Hence, simultaneous PIM and FLT3 inhibition represents a promising strategy in AML therapy. Treating SCID/beige mice bearing MV-4-11 tumors with SEL24-B489 results in a marked dose-dependent tumor reduction (67%, 74%, and 82% tumor growth inhibition for 50, 75, and 100 mg/kg daily doses, respectively) [61]. Despite promising results, there is no ongoing clinical trial investigating the potential of SEL24-B489.

In addition to the development of improved monotherapies for mutated FLT3, combining currently approved FLT3 inhibitors with the BCL-2 inhibitor, venetoclax, has shown promising results [62]. The combination of FLT3 inhibitors and BCL-2 inhibitors is particularly exciting for AML patients with comorbidities that limit the use of intensive induction chemotherapy, including patients over the age of 75 years. Venetoclax, in combination with hypomethylating agents, was FDA-approved in 2018 for these patients. Resistance to venetoclax may develop through increasing antiapoptotic proteins, including BCL-2 and myeloid cell leukemia 1 (MCL-1). However, data demonstrated a reciprocal benefit of combining FLT3 inhibitors with BCL-2 inhibitors. Both venetoclax and constitutively active FLT3 signaling increased MCL-1 expression and sequestration of the proapoptotic protein BIM [63,64], and the FLT3 inhibitors midostaurin and gilteritinib reduced the venetoclax-induced expression of MCL-1 [65]. Both midostaurin and gilteritinib synergized with venetoclax, increasing cell death in MOLM-13 [65,66], MOLM-14 [67], and MV-4-11 [65-67] cell lines. Singh Mali et al. comprehensively demonstrated efficacy and mechanisms of venetoclax combined with quizartinib in preclinical models of AML using MV-4-11 and MOLM-13 cells [68]. To our Knowledge, FLT3 inhibitor and venetoclax combinations have not been tested on other FLT3-mutated AML cell lines. There are some negative effects of venetoclax and FLT3 inhibitor combinations on nontransformed cells, and further research and development will be required before using this combination to treat patients with minimal side effects. Altering the balance between proapoptotic proteins and prosurvival proteins continues to be a logical goal in treating AML.

## Conclusions

Only a few human myeloid leukemia cell lines express mutated FLT3, despite approximately one-third of patients with AML having a mutation in FLT3. Despite different genetics, these cell lines are susceptible to drugs that inhibit FLT3 signaling and resistance can be minimized with combinations of FLT3 inhibition and BCL-2 inhibition. Continued collaborative research is needed to define the molecular and cytogenetic events, including germ line mutations, that initiate the cascade of events that result in AML, the molecular events that result in resistance to current therapies, and therapeutic strategies to overcome AML resistance. Collaborative research would increase the pool of mutated FLT3 mutated cell lines available for basic science research. Continued advances in the molecular understanding of AML will result in improved precision and personalized medicine. These advances are possible through collaborative efforts between basic science researchers and physicians.

## References

[1] Forsythe A, Sandman K (2021). What does the economic burden of acute myeloid leukemia treatment look like for the next decade? An analysis of key findings, challenges and recommendations. J Blood Med.

[2] Shallis RM, Wang R, Davidoff A, Ma X, Zeidan AM (2019). Epidemiology of acute myeloid leukemia: recent progress and enduring challenges. Blood Rev.

[3] Eden RE, Coviello JM (2023). Chronic Myelogenous Leukemia. http://www.ncbi.nlm.nih.gov/books/NBK531459/.

[4] Sampaio MM, Santos ML, Marques HS (2021). Chronic myeloid leukemia-from the Philadelphia chromosome to specific target drugs: a literature review. World J Clin Oncol.

[5] Döhner H, Estey E, Grimwade D (2017). Diagnosis and management of AML in adults: 2017 ELN recommendations from an international expert panel. Blood.

[6] Chennamadhavuni A, Lyengar V, Mukkamalla SKR, Shimanovsky A (2023). Leukemia. https://www.ncbi.nlm.nih.gov/books/NBK560490/.

[7] Kiyoi H, Naoe T, Yokota S (1997). Internal tandem duplication of FLT3 associated with leukocytosis in acute promyelocytic leukemia. Leukemia.

[8] Meshinchi S, Woods WG, Stirewalt DL (2001). Prevalence and prognostic significance of Flt3 internal tandem duplication in pediatric acute myeloid leukemia. Blood.

[9] Nakao M, Yokota S, Iwai T (1996). Internal tandem duplication of the FLT3 gene found in acute myeloid leukemia. Leukemia.

[10] Yokota S, Kiyoi H, Nakao M (1997). Internal tandem duplication of the FLT3 gene is preferentially seen in acute myeloid leukemia and myelodysplastic syndrome among various hematological malignancies. A study on a large series of patients and cell lines. Leukemia.

[11] Fernandez S, Desplat V, Villacreces A (2019). Targeting tyrosine kinases in acute myeloid leukemia: why, who and how?. Int J Mol Sci.

[12] Abu-Duhier FM, Goodeve AC, Wilson GA, Care RS, Peake IR, Reilly JT (2001). Identification of novel FLT-3 Asp835 mutations in adult acute myeloid leukaemia. Br J Haematol.

[13] Yamamoto Y, Kiyoi H, Nakano Y (2001). Activating mutation of D835 within the activation loop of FLT3 in human hematologic malignancies. Blood.

[14] Kazi JU, Rönnstrand L (2019). FMS-like tyrosine kinase 3/FLT3: from basic science to clinical implications. Physiol Rev.

[15] Takahashi S (2011). Downstream molecular pathways of FLT3 in the pathogenesis of acute myeloid leukemia: biology and therapeutic implications. J Hematol Oncol.

[16] Quentmeier H, Reinhardt J, Zaborski M, Drexler HG (2003). FLT3 mutations in acute myeloid leukemia cell lines. Leukemia.

[17] DepMap 23Q4 Public (2025). DepMap 25Q2 public. https://depmap.org/portal/data_page/?tab=allData.

[18] Ghandi M, Huang FW, Jané-Valbuena J (2019). Next-generation characterization of the cancer cell line encyclopedia. Nature.

[19] Spiekermann K, Dirschinger RJ, Schwab R (2003). The protein tyrosine kinase inhibitor SU5614 inhibits FLT3 and induces growth arrest and apoptosis in AML-derived cell lines expressing a constitutively activated FLT3. Blood.

[20] Ziegler-Heitbrock HW, Thiel E, Fütterer A, Herzog V, Wirtz A, Riethmüller G (1988). Establishment of a human cell line (Mono Mac 6) with characteristics of mature monocytes. Int J Cancer.

[21] Steube KG, Teepe D, Meyer C, Zaborski M, Drexler HG (1997). A model system in haematology and immunology: the human monocytic cell line MONO-MAC-1. Leuk Res.

[22] Smardová J, Pavlová S, Svitáková M, Grochová D, Ravcuková B (2005). Analysis of p53 status in human cell lines using a functional assay in yeast: detection of new non-sense p53 mutation in codon 124. Oncol Rep.

[23] Gerritsen M, Hilgendorf S, Yi G, Wierenga AT, Schuringa JJ, Martens JH, Vellenga E (2022). Presence of mutant p53 increases stem cell frequency and is associated with reduced binding to classic TP53 binding sites in cell lines and primary AMLs. Exp Hematol.

[24] Matsuo Y, MacLeod RA, Uphoff CC (1997). Two acute monocytic leukemia (AML-M5a) cell lines (MOLM-13 and MOLM-14) with interclonal phenotypic heterogeneity showing MLL-AF9 fusion resulting from an occult chromosome insertion, ins(11;9)(q23;p22p23). Leukemia.

[25] Asou H, Gombart AF, Takeuchi S (2003). Establishment of the acute myeloid leukemia cell line Kasumi-6 from a patient with a dominant-negative mutation in the DNA-binding region of the C/EBPalpha gene. Genes Chromosomes Cancer.

[26] Banker DE, Radich J, Becker A, Kerkof K, Norwood T, Willman C, Appelbaum FR (1998). The t(8;21) translocation is not consistently associated with high Bcl-2 expression in de novo acute myeloid leukemias of adults. Clin Cancer Res.

[27] Lange B, Valtieri M, Santoli D (1987). Growth factor requirements of childhood acute leukemia: establishment of GM-CSF-dependent cell lines. Blood.

[28] Ping N, Qiu H, Wang Q (2015). Establishment and genetic characterization of a novel mixed-phenotype acute leukemia cell line with EP300-ZNF384 fusion. J Hematol Oncol.

[29] Zhao X, Wang P, Diedrich JD (2022). Epigenetic activation of the FLT3 gene by ZNF384 fusion confers a therapeutic susceptibility in acute lymphoblastic leukemia. Nat Commun.

[30] Kennedy VE, Smith CC (2020). FLT3 mutations in acute myeloid leukemia: key concepts and emerging controversies. Front Oncol.

[31] Larrosa-Garcia M, Baer MR (2017). FLT3 inhibitors in acute myeloid leukemia: current status and future directions. Mol Cancer Ther.

[32] Mori M, Kaneko N, Ueno Y (2017). Gilteritinib, a FLT3/AXL inhibitor, shows antileukemic activity in mouse models of FLT3 mutated acute myeloid leukemia. Invest New Drugs.

[33] Daver N, Venugopal S, Ravandi F (2021). FLT3 mutated acute myeloid leukemia: 2021 treatment algorithm. Blood Cancer J.

[34] Yang W, Soares J, Greninger P (2013). Genomics of Drug Sensitivity in Cancer (GDSC): a resource for therapeutic biomarker discovery in cancer cells. Nucleic Acids Res.

[35] (2025). Genomics of drug sensitivity in cancer, version 8.5. http://www.cancerrxgene.org.

[36] Stone RM, Mandrekar SJ, Sanford BL (2017). Midostaurin plus chemotherapy for acute myeloid leukemia with a FLT3 mutation. N Engl J Med.

[37] Tzogani K, Yu Y, Meulendijks D (2019). European Medicines Agency review of midostaurin (Rydapt) for the treatment of adult patients with acute myeloid leukaemia and systemic mastocytosis. ESMO Open.

[38] Lee LY, Hernandez D, Rajkhowa T (2017). Preclinical studies of gilteritinib, a next-generation FLT3 inhibitor. Blood.

[39] Dumas PY, Villacreces A, Guitart AV (2021). Dual inhibition of FLT3 and AXL by gilteritinib overcomes hematopoietic niche-driven resistance mechanisms in FLT3-ITD acute myeloid leukemia. Clin Cancer Res.

[40] Levis M (2014). Quizartinib for the treatment of FLT3/ITD acute myeloid leukemia. Future Oncol.

[41] Röllig C, Serve H, Noppeney R (2021). Sorafenib or placebo in patients with newly diagnosed acute myeloid leukaemia: long-term follow-up of the randomized controlled SORAML trial. Leukemia.

[42] Burchert A, Bug G, Fritz LV (2020). Sorafenib maintenance after allogeneic hematopoietic stem cell transplantation for acute myeloid leukemia with FLT3-internal tandem duplication mutation (SORMAIN). J Clin Oncol.

[43] Blackmon A, Aldoss I, Ball BJ (2022). FLT3 inhibitors as maintenance therapy after allogeneic stem-cell transplantation. Blood Lymphat Cancer.

[44] Zimmerman EI, Turner DC, Buaboonnam J (2013). Crenolanib is active against models of drug-resistant FLT3-ITD-positive acute myeloid leukemia. Blood.

[45] Galanis A, Ma H, Rajkhowa T, Ramachandran A, Small D, Cortes J, Levis M (2014). Crenolanib is a potent inhibitor of FLT3 with activity against resistance-conferring point mutants. Blood.

[46] Scholl S, Fleischmann M, Schnetzke U, Heidel FH (2020). Molecular mechanisms of resistance to FLT3 inhibitors in acute myeloid leukemia: ongoing challenges and future treatments. Cells.

[47] Daver N, Cortes J, Ravandi F, Patel KP, Burger JA, Konopleva M, Kantarjian H (2015). Secondary mutations as mediators of resistance to targeted therapy in leukemia. Blood.

[48] Cools J, Mentens N, Furet P (2004). Prediction of resistance to small molecule FLT3 inhibitors: implications for molecularly targeted therapy of acute leukemia. Cancer Res.

[49] Zhang H, Savage S, Schultz AR (2019). Clinical resistance to crenolanib in acute myeloid leukemia due to diverse molecular mechanisms. Nat Commun.

[50] McMahon CM, Ferng T, Canaani J (2019). Clonal selection with RAS pathway activation mediates secondary clinical resistance to selective FLT3 inhibition in acute myeloid leukemia. Cancer Discov.

[51] Chang Y-T, Hernandez D, Ghiaur G, Levis MJ, Jones RJ (2017). Bone marrow stroma protects FLT3 acute myeloid leukemia (AML) through CYP3A4-mediated drug metabolization of FLT3 tyrosine kinase inhibitors (TKIs). Blood.

[52] Sato T, Yang X, Knapper S (2011). FLT3 ligand impedes the efficacy of FLT3 inhibitors in vitro and in vivo. Blood.

[53] Traer E, Martinez J, Javidi-Sharifi N (2016). FGF2 from marrow microenvironment promotes resistance to FLT3 inhibitors in acute myeloid leukemia. Cancer Res.

[54] Yang X, Sexauer A, Levis M (2014). Bone marrow stroma-mediated resistance to FLT3 inhibitors in FLT3-ITD AML is mediated by persistent activation of extracellular regulated kinase. Br J Haematol.

[55] Maraskovsky E, Brasel K, Teepe M, Roux ER, Lyman SD, Shortman K, McKenna HJ (1996). Dramatic increase in the numbers of functionally mature dendritic cells in FLT3 ligand-treated mice: multiple dendritic cell subpopulations identified. J Exp Med.

[56] Maraskovsky E, Daro E, Roux E (2000). In vivo generation of human dendritic cell subsets by Flt3 ligand. Blood.

[57] Rickmann M, Krauter J, Stamer K (2011). Elevated frequencies of leukemic myeloid and plasmacytoid dendritic cells in acute myeloid leukemia with the FLT3 internal tandem duplication. Ann Hematol.

[58] Ferng TT, Terada D, Ando M (2022). The irreversible FLT3 inhibitor FF-10101 is active against a diversity of FLT3 inhibitor resistance mechanisms. Mol Cancer Ther.

[59] Yamaura T, Nakatani T, Uda K (2018). A novel irreversible FLT3 inhibitor, FF-10101, shows excellent efficacy against AML cells with FLT3 mutations. Blood.

[60] Pratz KW, Kaplan J, Levy M (2023). A phase Ib trial of mivavotinib (TAK-659), a dual SYK/FLT3 inhibitor, in patients with relapsed/refractory acute myeloid leukemia. Haematologica.

[61] Czardybon W, Windak R, Gołas A (2018). A novel, dual pan-PIM/FLT3 inhibitor SEL24 exhibits broad therapeutic potential in acute myeloid leukemia. Oncotarget.

[62] Wei AH, Roberts AW (2023). BCL2 inhibition: a new paradigm for the treatment of AML and beyond. Hemasphere.

[63] Niu X, Zhao J, Ma J (2016). Binding of released Bim to Mcl-1 is a mechanism of intrinsic resistance to ABT-199 which can be overcome by combination with daunorubicin or cytarabine in AML cells. Clin Cancer Res.

[64] Yoshimoto G, Miyamoto T, Jabbarzadeh-Tabrizi S (2009). FLT3-ITD up-regulates MCL-1 to promote survival of stem cells in acute myeloid leukemia via FLT3-ITD-specific STAT5 activation. Blood.

[65] Ma J, Zhao S, Qiao X (2019). Inhibition of Bcl-2 synergistically enhances the antileukemic activity of midostaurin and gilteritinib in preclinical models of FLT3-mutated acute myeloid leukemia. Clin Cancer Res.

[66] Brinton LT, Zhang P, Williams K (2020). Synergistic effect of BCL2 and FLT3 co-inhibition in acute myeloid leukemia. J Hematol Oncol.

[67] Zhu R, Li L, Nguyen B (2021). FLT3 tyrosine kinase inhibitors synergize with BCL-2 inhibition to eliminate FLT3/ITD acute leukemia cells through BIM activation. Signal Transduct Target Ther.

[68] Singh Mali R, Zhang Q, DeFilippis RA (2021). Venetoclax combines synergistically with FLT3 inhibition to effectively target leukemic cells in FLT3-ITD+ acute myeloid leukemia models. Haematologica.

